# Epidermoid Cyst of the Cecum: A Case Report of an Exceptionally Rare Entity and Review of the Literature

**DOI:** 10.7759/cureus.117646

**Published:** 2026-10-08

**Authors:** Nikolaos P Fotiou, Iraklis Evangelou Lampi, Christina Dastamani, Kostas Sotirios Karakantas, Athanasios Deliveliotis

**Affiliations:** 1 Department of General Surgery, General Hospital of Karditsa, Karditsa, GRC; 2 Department of Pathology, General Hospital of Karditsa, Karditsa, GRC

**Keywords:** appendiceal mucocele, cecal epidermoid cyst, epidermoid cyst, right hemicolectomy, right lower quadrant mass

## Abstract

Epidermoid cysts of the cecum are extremely rare benign lesions of the gastrointestinal tract. Their diagnosis remains challenging due to the lack of specific clinical symptoms and inconclusive radiological findings. Consequently, accurate preoperative diagnosis is difficult, with lesions often being misdiagnosed as appendiceal mucoceles, gastrointestinal stromal tumors (GISTs), duplication cysts, or adnexal masses. We report the case of a 59-year-old male who presented with right lower quadrant abdominal pain and a palpable mass. Comprehensive diagnostic imaging, including abdominal ultrasonography, abdominopelvic computed tomography, and magnetic resonance imaging (MRI), revealed a well-circumscribed cystic lesion arising from the cecal wall, initially concerning for a cecal neoplasm or appendiceal mucocele. The patient underwent a successful right hemicolectomy, and histopathological examination definitively confirmed a cecal epidermoid cyst lined by stratified squamous epithelium containing keratinaceous debris. Postoperative recovery was uneventful. Although exceptionally rare, epidermoid cysts should be included in the differential diagnosis of right lower quadrant masses. Despite the limited number of reported cases, complete surgical excision remains the definitive treatment of choice, providing an excellent long-term prognosis with no evidence of recurrence.

## Introduction

Epidermoid cysts are benign lesions lined by stratified squamous epithelium that are most commonly encountered in the skin and subcutaneous tissues. Although they have been described in several visceral organs, including the spleen, liver, kidney, and testis, involvement of the cecum remains exceptionally rare [1]. The exact pathogenesis of cecal epidermoid cysts is still unclear. Both congenital and acquired mechanisms have been proposed, including ectodermal sequestration during embryological development and iatrogenic implantation of epithelial elements following abdominal surgery or trauma [2].

To date, only a limited number of cases have been reported in the English-language literature. Because of their rarity, the clinical characteristics and optimal management of these lesions remain poorly defined. Patients usually present with non-specific symptoms, while radiological findings frequently resemble other cystic or neoplastic lesions of the ileocecal region. As a result, establishing a definitive preoperative diagnosis is often difficult, and most cases are diagnosed only after surgical resection and histopathological examination [3].

We report the case of a 59-year-old man with an epidermoid cyst of the cecum who underwent right hemicolectomy. In addition, we review the available English-language literature and summarize the clinical presentation, diagnostic workup, histopathological features, and surgical management of this exceptionally rare entity.

## Case presentation

A 59-year-old man was referred to our outpatient surgical clinic with a two-month history of non-specific abdominal pain, abdominal fullness, and a progressively enlarging palpable mass in the right iliac fossa. Physical examination confirmed the presence of a non-tender palpable mass in the right lower quadrant, while the remainder of the abdominal examination was unremarkable. His medical history was significant for hypertension and dyslipidemia, both adequately controlled with medical treatment. There was no history of previous abdominal surgery, abdominal trauma, or chronic inflammatory bowel disease. Routine laboratory investigations, including inflammatory markers, were within normal limits, and no evidence of anemia was identified. Serum tumor markers were also within the normal reference range.

Abdominal ultrasonography demonstrated a large, well-circumscribed cystic lesion in the right lower quadrant. The lesion showed predominantly homogeneous low-level internal echoes, suggestive of proteinaceous or keratinaceous content, and measured approximately 12 cm in maximum diameter. Color Doppler examination demonstrated no internal vascularity. The lesion appeared closely related to the cecal region, although its exact origin could not be determined, as demonstrated in Figure 1. Magnetic resonance imaging (MRI) demonstrated a well-defined cystic mass measuring approximately 12 cm in maximum diameter within the right iliac fossa, closely related to the cecum. The lesion exhibited low-to-intermediate signal intensity on T1-weighted images and predominantly high signal intensity on T2-weighted images. Diffusion-weighted imaging demonstrated restricted diffusion within the lesion. No definite invasion of adjacent structures or regional lymphadenopathy was identified. Although the lesion appeared predominantly cystic, its exact nature could not be determined preoperatively, as shown in Figure 2. Colonoscopy was unremarkable, demonstrating normal appearances of the terminal ileum, ileocecal valve, and cecal mucosa, with no evidence of an intraluminal lesion. The main preoperative differential diagnoses included an appendiceal mucocele and a cecal cystic neoplasm, while malignancy could not be excluded.

**Figure 1:**
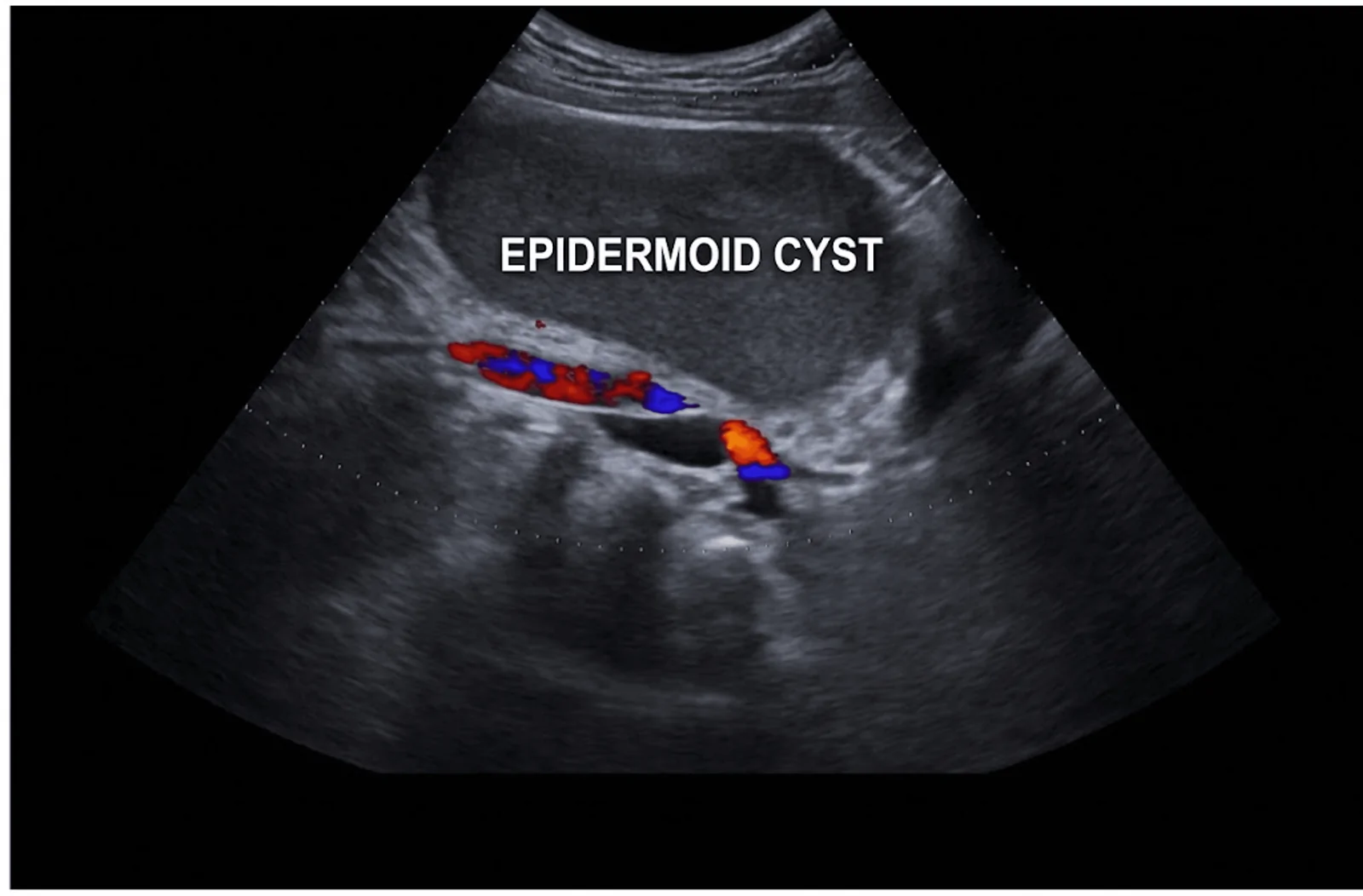
Transabdominal color Doppler ultrasonography of the cecal mass Transabdominal color Doppler ultrasound image demonstrating a well-defined, unilocular cystic lesion (labeled "EPIDERMOID CYST") located in the right lower quadrant, adjacent to the cecum. Color Doppler mode highlights peripheral vascular flow within the adjacent tissue/capsule without internal vascularity inside the cyst cavity.

**Figure 2:**
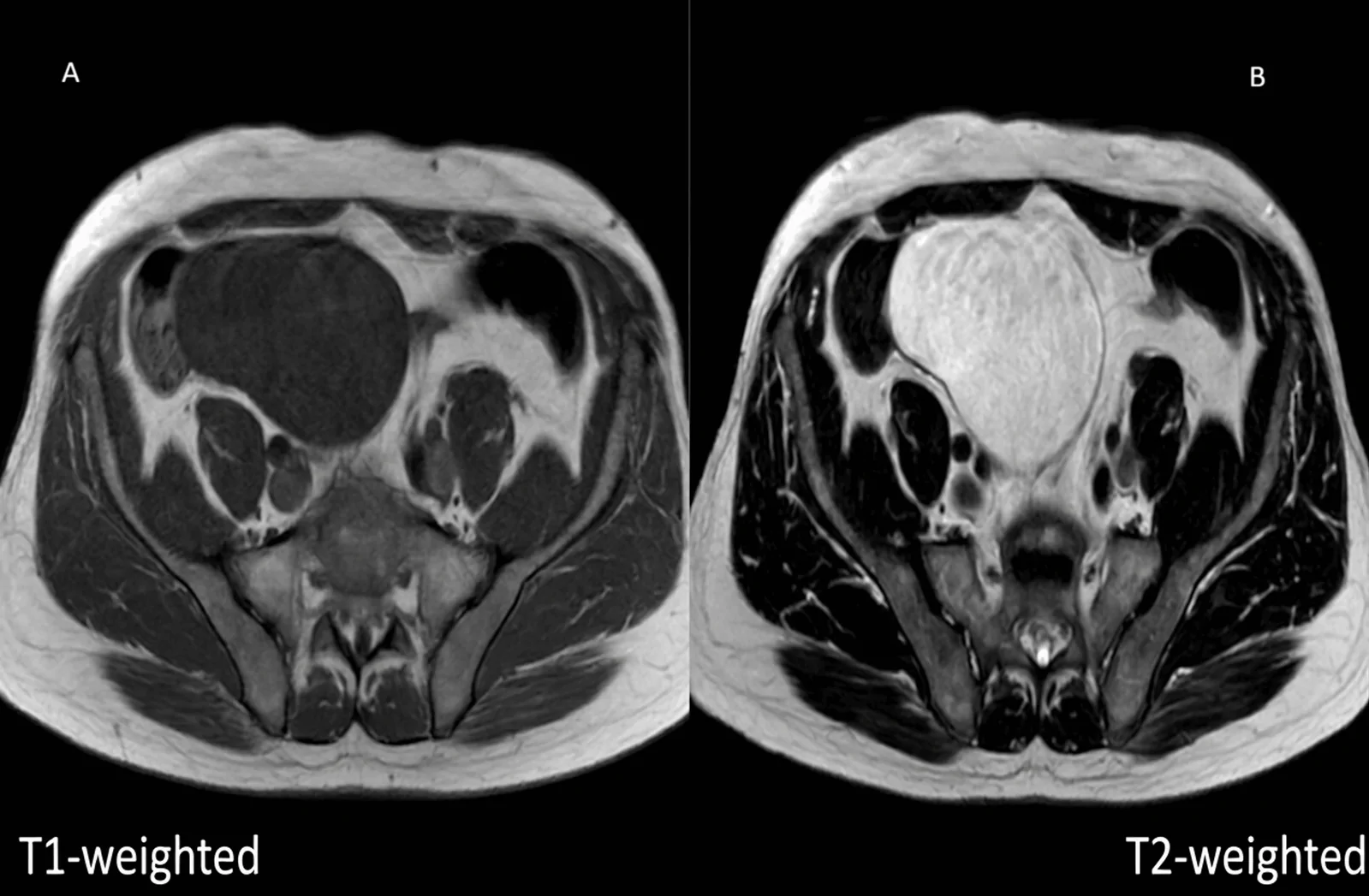
Axial MRI views of the cecal mass Compare the signal intensity of the subserosal cecal mass on different sequences. (A) T1-weighted image shows the cyst with low signal intensity (hypointense). (B) T2-weighted image shows the cyst with high signal intensity (hyperintense), consistent with fluid content.

Therefore, an elective exploratory laparotomy was scheduled. Intraoperatively, a large cystic mass was identified arising from the antimesenteric border of the cecum. The appendix could not be identified separately within the right iliac fossa. The lesion had a smooth external surface and appeared adherent to the surrounding adipose tissue. No evidence of infiltration into adjacent organs or regional lymphadenopathy was observed. The cecum and terminal ileum appeared macroscopically unremarkable. Given the preoperative suspicion of a neoplastic lesion and the intraoperative findings, a right hemicolectomy with ileotransverse anastomosis was performed, as depicted in Figure 3. The postoperative course was uneventful, with no early postoperative complications. Bowel function recovered normally, and oral intake was gradually resumed without difficulty. The patient was discharged home on the fifth postoperative day in good clinical condition.

**Figure 3:**
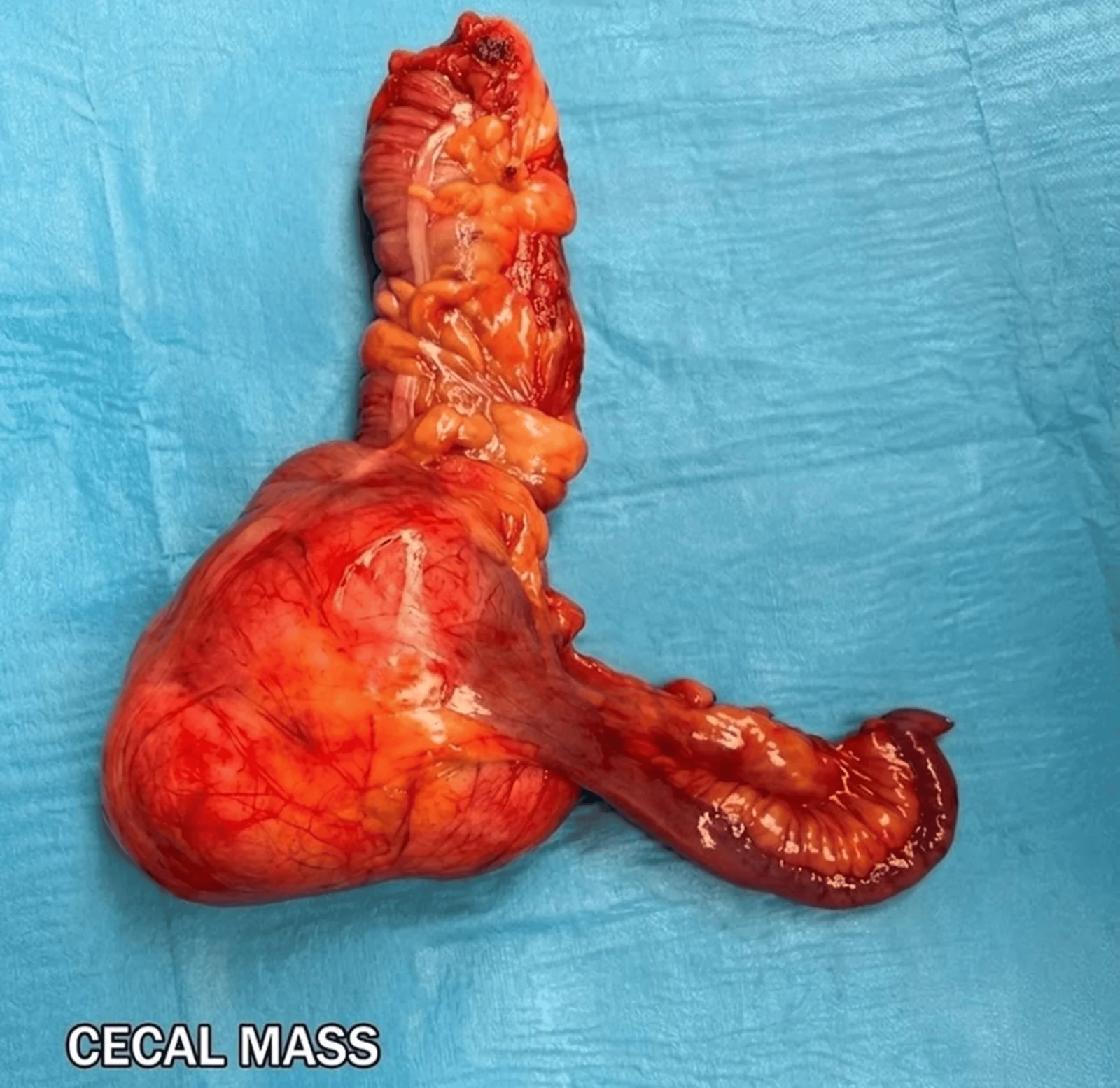
Gross pathology of the resected specimen Macroscopic view of the surgical specimen showing the cecal mass arising from the cecum.

Definitive diagnosis of the cecal epidermoid cyst was achieved following histopathological examination of the resected specimen. Gross pathological examination demonstrated a cystic lesion of the cecum measuring 12 cm in maximum diameter, with a relatively smooth external surface and a cavity filled with whitish friable keratinaceous material, as presented in Figure 4. Microscopically, the cyst wall was composed of loose fibrous tissue lined by mature keratinizing stratified squamous epithelium with a prominent granular layer, while the cavity contained keratinaceous debris. Smooth muscle bundles originating from the outer longitudinal layer of the cecal muscularis propria were identified on the external surface of the cyst wall. This histological finding aligns with the theory of a congenital origin regarding the development of cecal epidermoid cysts. The histopathological features are illustrated in Figures 5, 6. The appendix showed fibrous obliteration of its lumen, while the terminal ileum demonstrated reactive lymphoid hyperplasia. Nineteen regional lymph nodes were retrieved and exhibited predominantly reactive lymphoid hyperplasia without evidence of malignancy. No significant microscopic abnormalities were identified in the adjacent colonic mucosa, and all resection margins were free of pathological involvement.

**Figure 4:**
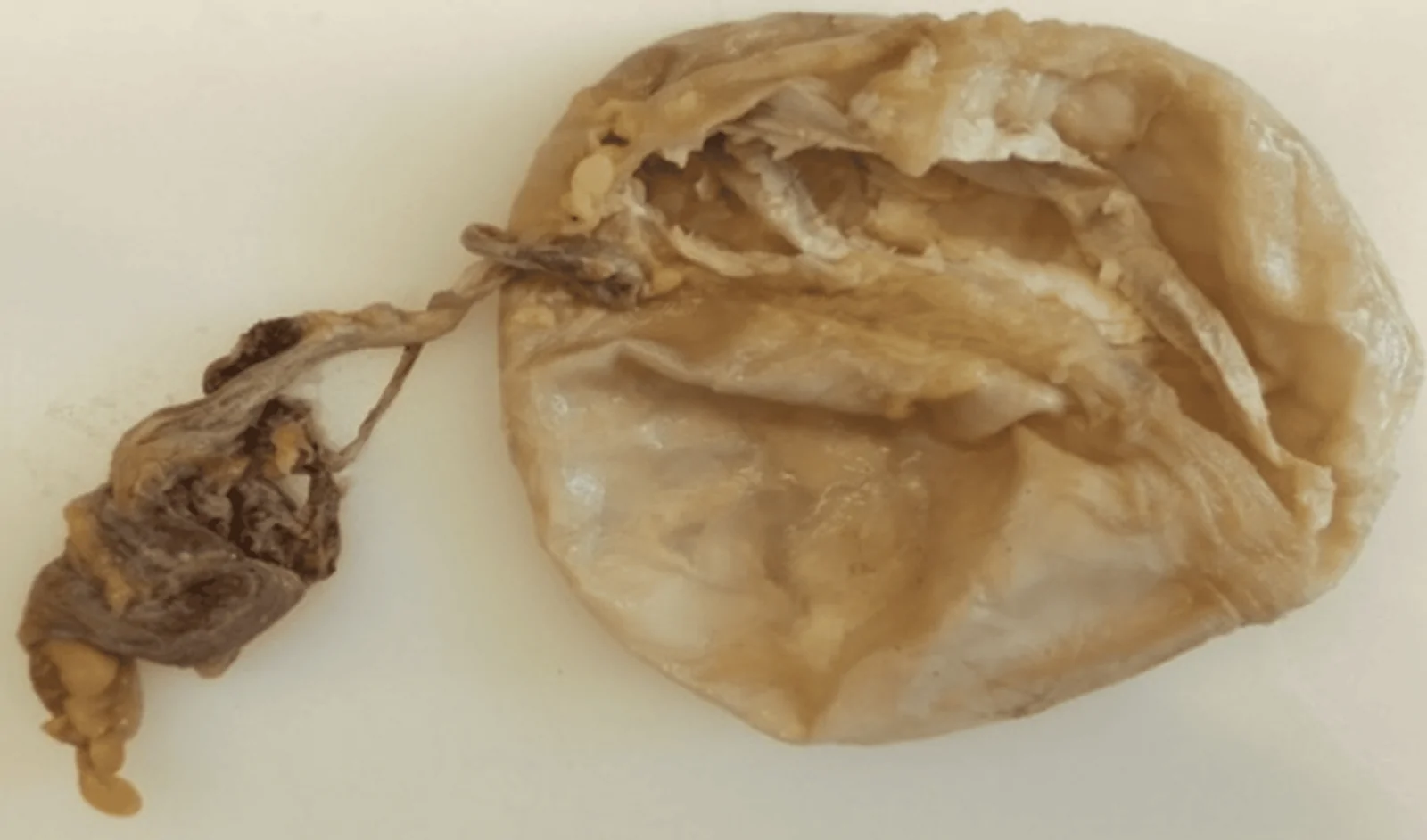
Gross pathology of the excised cyst Macroscopic view of the resected cecal epidermoid cyst following formalin fixation.

**Figure 5:**
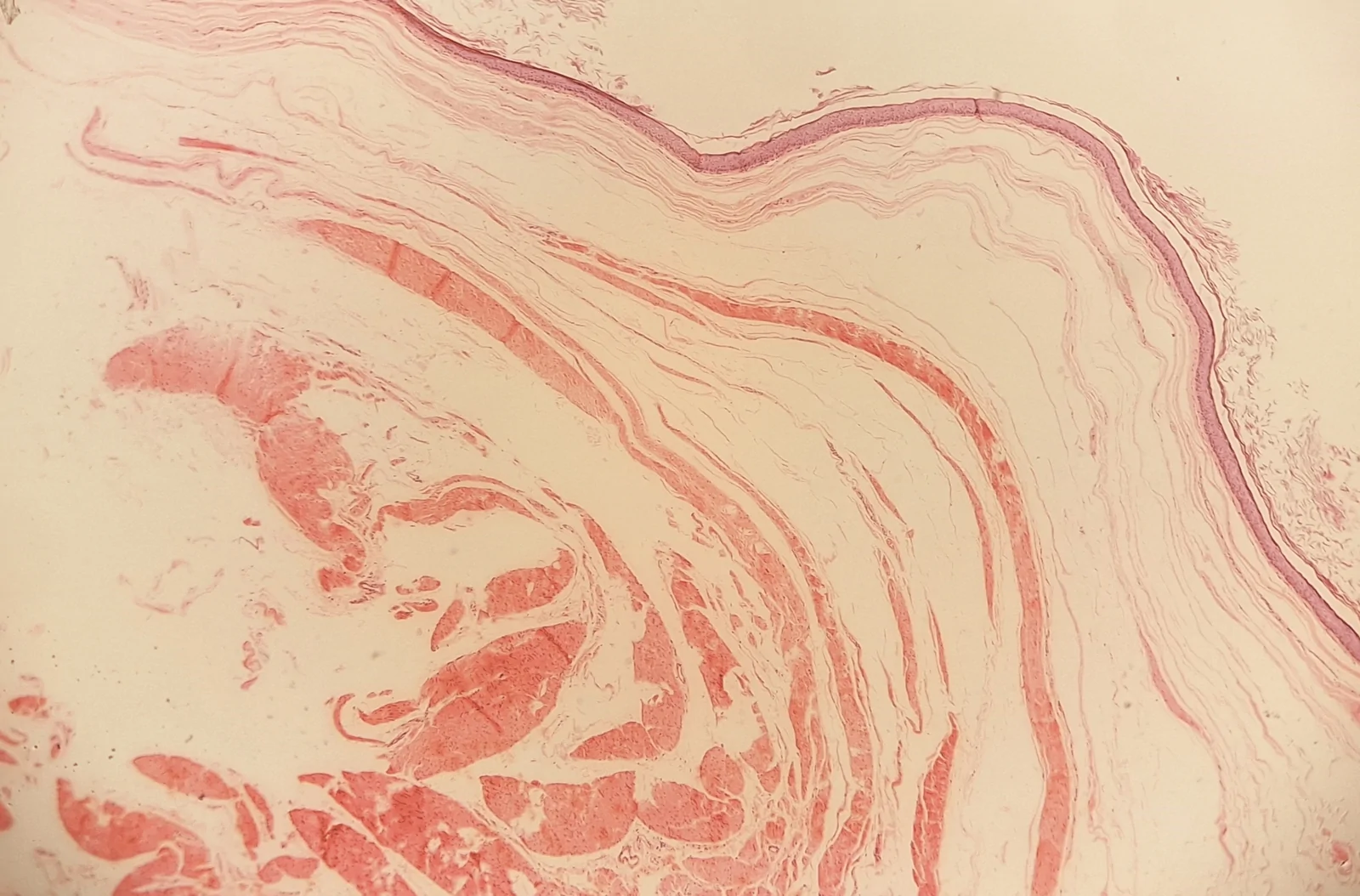
Histology of the cecal cyst wall and muscularis propria Microscopic image showing the cyst wall in close association with smooth muscle bundles of the outer longitudinal layer of the cecal muscularis propria. H&E, ×25.

**Figure 6:**
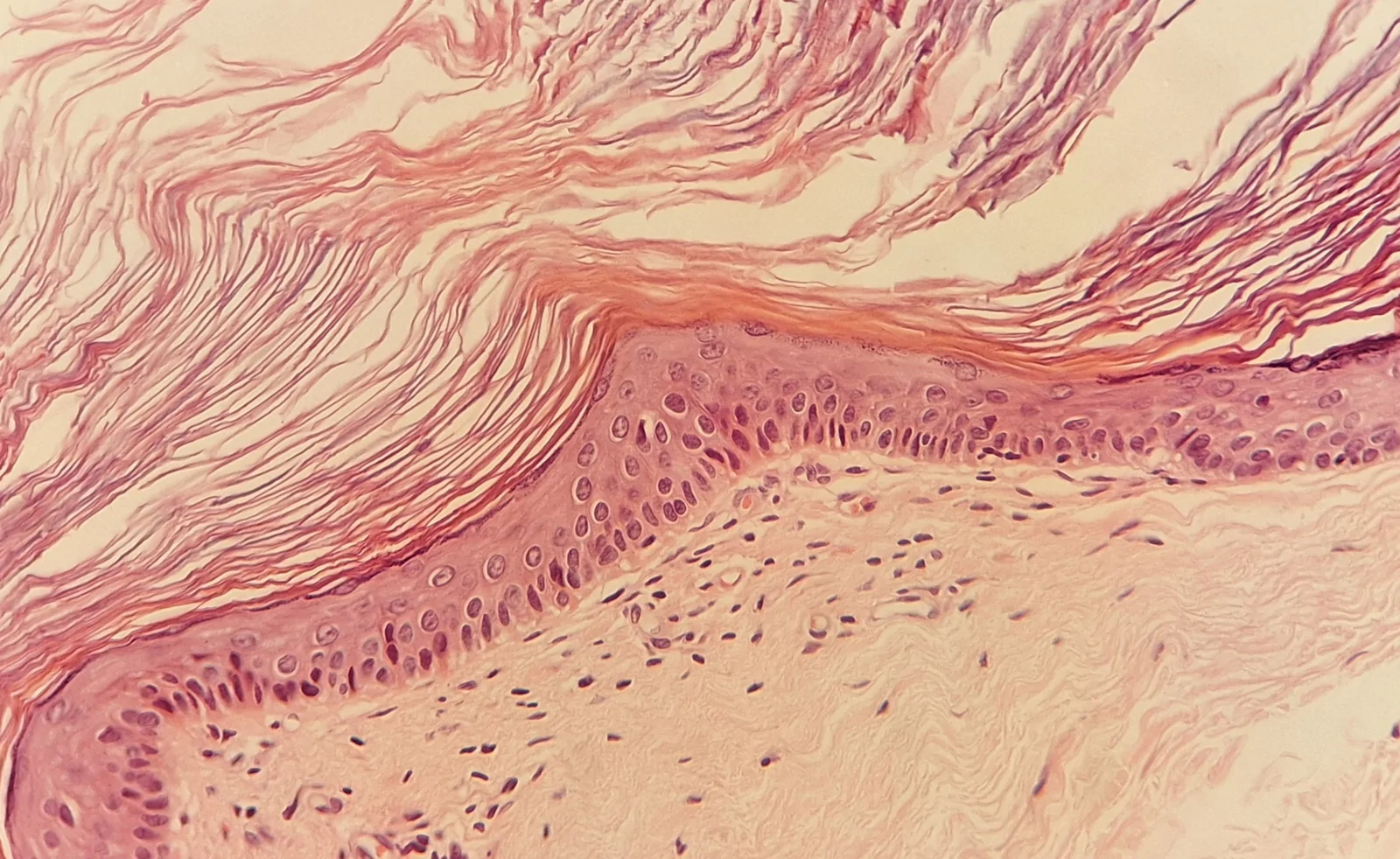
Histopathology showing keratinized stratified squamous epithelial lining The cyst was lined by keratinized stratified squamous epithelium with a granular layer. Skin adnexal structures were completely absent. H&E, ×200.

## Discussion

Epidermoid cysts of the cecum represent an extremely rare condition. Although epidermoid cysts may occur in various anatomical locations, including the spleen, liver, testes, kidneys, and appendix, involvement of the cecum is exceptionally uncommon [1]. The histopathogenesis of cecal epidermoid cysts remains unclear. These lesions are generally considered sequestration cysts and may be classified as either congenital or acquired [4].

The exact histogenesis of congenital cecal epidermoid cysts remains controversial, as the cecum is embryologically derived from the endoderm, whereas epidermoid cysts are lined by stratified squamous epithelium of ectodermal origin. Two principal embryological theories have been proposed. The most widely accepted hypothesis suggests aberrant sequestration of ectodermal elements during embryogenesis. This mechanism is closely related to physiological midgut herniation and subsequent intestinal rotation. During re-entry of the midgut into the abdominal cavity and fixation of the cecum in the right iliac fossa, ectodermal cells may become entrapped within the subserosal or intramural layers of the cecal wall. Progressive proliferation of these ectopic cells and accumulation of keratin may subsequently result in cyst formation [5]. An alternative theory proposes heterotopic differentiation or squamous metaplasia of pluripotent endodermal cells within the intestinal wall, leading to the development of squamous epithelium without ectodermal displacement [6]. Acquired epidermoid cysts are thought to result from iatrogenic implantation of epidermal elements during previous abdominal surgery or following abdominal trauma. Surgical manipulation may lead to displacement of epithelial cells into deeper tissues, where they gradually proliferate and form cystic lesions. Chronic inflammation has also been proposed as a possible contributing factor. Persistent inflammatory stimulation within the cecal wall may induce squamous metaplasia of entrapped pluripotent cells, eventually leading to cyst development; however, evidence supporting this mechanism remains limited [5,7].

To our knowledge, to date, only 15 cases of cecal epidermoid cysts have been reported in the English-language literature. The clinical characteristics, diagnostic findings, surgical strategies, and outcomes of all published cases, alongside the current report, are summarized in Table 1. The reported cases encompass both pediatric and adult populations, demonstrating a wide age spectrum. In four reported cases, a history of previous abdominal surgery was documented, supporting a possible acquired origin. Specifically, two of the earliest reported cases, published in 1961 and 1969, involved patients with a history of prior appendectomy [7,8]. More recently, two additional cases described patients with prior cesarean sections [9,10]. In the remaining reported cases, no history of abdominal surgery, abdominal trauma, or chronic inflammatory disease was identified, supporting a possible congenital origin. Similarly, our patient had no previous history of abdominal surgery or abdominal trauma, suggesting a congenital cecal epidermoid cyst.

**Table 1:** Clinical characteristics, diagnostic findings, and surgical management of published cases of cecal epidermoid cysts F: female; M: male; GIST: gastrointestinal stromal tumor

Author, Year	Age/Sex	Symptoms	Relevant History	Preoperative Diagnosis	Surgical Treatment	Outcome
							Pan, 1961 [8]	22 / F	Palpable mass	Previous appendectomy	Suspected cecal neoplasm	Right hemicolectomy	Uneventful
							Candreviotis, 1965 [11]	31 / F	Right lower quadrant pain	No prior abdominal surgery	Ovarian cyst/torsion	Ileocecal resection	Uneventful
							Pear, 1969 [7]	41 / M	Palpable mass	Previous appendectomy	Cecal neoplasm/abscess	Mass excision	Uneventful
Andiran, 1999 [5]	8 / F	Abdominal pain	No prior abdominal surgery	Cystic mass	Excision and appendectomy	Uneventful
Mady, 2002 [6]	67 / M	Nausea, vomiting, abdominal pain	No prior abdominal surgery	Duplication cyst	Right hemicolectomy	Uneventful
Uzunlar, 2006 [1]	75 / M	Nausea, vomiting, abdominal pain	No prior abdominal surgery	Appendiceal mucocele	Right hemicolectomy	Postoperative death
Demir, 2012 [9]	31 / F	Inguinal pain	Previous cesarean section	Adnexal mass	Mass excision	Uneventful
Sahoo, 2013 [4]	54 / M	Right lower quadrant pain, palpable mass	No prior abdominal surgery	Mesenteric cyst/GIST/appendicular lump	Mass excision	Uneventful
Mittal, 2015 [12]	25 / F	Abdominal pain, pelvic mass	No prior abdominal surgery	Intestinal tumor	Right hemicolectomy	Uneventful
Park, 2015 [13]	63 / F	Asymptomatic	No prior abdominal surgery	GIST	Ileocecal resection	Uneventful
Tonelli, 2021 [14]	51 / M	Asymptomatic	No prior abdominal surgery	Appendiceal mucocele	Ileocecal resection	Uneventful
Tominaga, 2021 [3]	20 / F	Abdominal pain	No prior abdominal surgery	GIST/duplication cyst	Ileocecal resection	Uneventful
Hfaiedh, 2024 [2]	38 / F	Pelvic discomfort	No prior abdominal surgery	Adnexal mass	Right hemicolectomy	Uneventful
Firrincieli, 2025 [15]	28 / F	Abdominal pain	No prior abdominal surgery	Duplication cyst/endometriotic cyst	Right hemicolectomy	Uneventful
Keating, 2026 [10]	35 / F	Dysmenorrhea, pelvic pain	Previous cesarean section	Intramuscular cecal mass	Right hemicolectomy	Uneventful
Present case, 2026	59 / M	Abdominal pain, palpable mass	No prior abdominal surgery	Cecal neoplasm	Right hemicolectomy	Uneventful
				Appendiceal mucocele		

Most patients with epidermoid cysts of the cecum present with non-specific symptoms, including abdominal pain, nausea, vomiting, or abdominal fullness, while some cases are associated with a palpable abdominal mass [2,6,11]. In female patients, gynecological symptoms have also been reported, occasionally leading to confusion with adnexal pathology [2,10,12]. Only a limited number of reported cases presented with recent-onset abdominal pain, with symptom duration not exceeding two weeks [1,5,12]. Notably, none of the reported patients presented with an acute abdomen. In several patients, the lesion was detected incidentally during radiological investigations performed for unrelated reasons [13,14]. Similarly, our patient presented only with non-specific abdominal symptoms.

Preoperative diagnosis of cecal epidermoid cysts remains highly challenging because both clinical presentation and imaging findings are non-specific. Radiological findings of cecal epidermoid cysts are non-distinctive and frequently mimic other cystic or neoplastic lesions of the ileocecal region. Ultrasonography usually demonstrates a well-circumscribed cystic lesion with internal echogenic debris or heterogeneous echoes related to keratinaceous material [9]. Computed tomography typically reveals a well-defined low-attenuation cystic mass, occasionally associated with peripheral or capsular enhancement [3,12]. MRI findings may vary. In most reported cases, the lesion demonstrates low-to-intermediate signal intensity on T1-weighted images and high signal intensity on T2-weighted images, although a heterogeneous appearance may also be observed. Diffusion restriction has additionally been described in lesions containing dense keratin material [2,10]. The use of fine-needle aspiration (FNA) in the diagnosis of cecal epidermoid cysts remains limited and has been reported in only one case in the literature. Aspiration yielded grayish-yellow material, while cytological examination demonstrated numerous anucleate and nucleated squamous cells [12]. However, given the limited experience reported in the literature, the diagnostic role of FNA remains uncertain. Consequently, definitive diagnosis continues to rely on histopathological examination of the resected specimen. Colonoscopy also has limited diagnostic value in cecal epidermoid cysts, as these lesions are typically located within or outside the bowel wall while the overlying mucosa remains intact. Nevertheless, it may be useful in excluding mucosal pathology and malignant colonic lesions during the preoperative evaluation [4,10,13]. Consequently, definitive diagnosis in all reported cases was established only after surgical excision and histopathological examination.

The differential diagnosis of cecal epidermoid cysts remains particularly challenging and includes appendiceal mucocele, gastrointestinal stromal tumor (GIST), mesenteric cyst, duplication cyst, and other cystic or neoplastic intra-abdominal lesions. In female patients, these lesions may additionally mimic a right adnexal cystic mass [9]. In our case, the preoperative differential diagnosis mainly included appendiceal mucocele and a neoplastic lesion.

Histopathological examination remains the gold standard for the diagnosis of cecal epidermoid cysts. The characteristic histological findings include a cyst wall lined by keratinizing stratified squamous epithelium and the presence of keratinaceous debris within the cyst cavity. The absence of skin adnexal structures is a key feature that distinguishes epidermoid cysts from dermoid cysts and other developmental cystic lesions [1]. In the present case, histopathological evaluation confirmed the diagnosis and excluded alternative cystic and neoplastic entities of the ileocecal region.

According to the available literature, right hemicolectomy represents the most commonly performed surgical procedure for epidermoid cysts of the cecum. This is mainly attributed to the difficulty of establishing a definitive preoperative diagnosis and the inability to exclude malignancy [2]. Complete surgical excision remains the treatment of choice, either by right hemicolectomy or isolated cyst excision, in order to establish a definitive diagnosis and prevent local recurrence. Only a limited number of cases treated with isolated cyst excision have been reported, with no recurrence described during follow-up [4,5,7,9]. In our patient, a right hemicolectomy with ileotransverse anastomosis was performed.

There is currently no established postoperative surveillance protocol for cecal epidermoid cysts due to the rarity of the condition. Nevertheless, long-term follow-up with laboratory and radiological evaluation has been recommended in the literature [2]. In the present case, follow-up colonoscopy and computed tomography performed three months postoperatively demonstrated no evidence of recurrence or other pathological findings. Tumor markers and routine laboratory investigations remained within normal limits. Among the 15 cases reported to date, including the present case, no recurrences have been observed [1-15].

## Conclusions

Cecal epidermoid cysts are exceptionally rare benign lesions, with only a limited number of cases reported in the English-language literature. Their clinical presentation is usually non-specific and ranges from incidental findings to abdominal pain, abdominal fullness, or a palpable mass, making preoperative diagnosis particularly challenging. Radiological investigations, including ultrasonography, computed tomography, and MRI, may identify a cystic lesion but are generally unable to establish a definitive diagnosis because of the overlap with other cystic and neoplastic conditions of the ileocecal region. Consequently, epidermoid cysts should be considered in the differential diagnosis of cystic masses arising from the cecum, especially when conventional investigations fail to provide a clear diagnosis.

Histopathological examination remains the gold standard for diagnosis, allowing differentiation from dermoid cysts and duplication cysts. Complete surgical excision, such as right hemicolectomy, is both diagnostic and therapeutic and represents the treatment of choice. Although no recurrence has been reported in the available literature, long-term follow-up may be considered because of the rarity of the condition and the limited available data. The present case further expands the existing literature and highlights the importance of considering this uncommon entity in the evaluation of cystic cecal masses.
